# An Improved Capacitive Sensor for Detecting the Micro-Clearance of Spherical Joints

**DOI:** 10.3390/s19122694

**Published:** 2019-06-14

**Authors:** Wen Wang, Wenjun Qiu, He Yang, Haimei Wu, Guang Shi, Zhanfeng Chen, Keqing Lu, Kui Xiang, Bingfeng Ju

**Affiliations:** 1 School of Mechanical Engineering, Hangzhou Dianzi University, Hangzhou 310018, China; wangwn@hdu.edu.cn (W.W.); qwjhdu@163.com (W.Q.); hellen0810@foxmail.com (H.W.); shiguang@hdu.edu.cn (G.S.); czf@hdu.edu.cn (Z.C.); lkq@hdu.edu.cn (K.L.); 2 School of Mechanical Engineering, Zhejiang University, Hangzhou 310027, China; snowboy2001102@163.com (K.X.); mbfju@zju.edu.cn (B.J.); 3 State Key Lab of Fluid Power & Mechatronic Systems, Zhejiang University, Hangzhou 310027, China

**Keywords:** spherical joint, clearance measurement, capacitive sensor, eccentric displacement

## Abstract

Due to the flexible and compact structures, spherical joints are widely used in parallel manipulators and industrial robots. Real-time detection of the clearance between the ball and the socket in spherical joints is beneficial to compensate motion errors of mechanical systems and improve their transmission accuracy. This work proposes an improved capacitive sensor for detecting the micro-clearance of spherical joints. First, the structure of the capacitive sensor is proposed. Then, the mathematical model for the differential capacitance of the sensor and the eccentric micro-displacement of the ball is deduced. Finally, the capacitance values of the capacitive sensor are simulated with Ansoft Maxwell. The simulated values of the differential capacitances at different eccentric displacements agree well with the theoretical ones, indicating the feasibility of the proposed detection method. In addition, the simulated results show that the proposed capacitive sensor could effectively reduce the capacitive fringe effect, improving the measurement accuracy.

## 1. Introduction

Due to its compact structure and flexible motion, precision spherical joints have been widely used in parallel manipulators and industrial robots [1]. In the traditional applications, spherical joints are usually assumed to be ideal and the clearance between the ball and the socket in spherical joints is ignored to simplify the dynamic model of the multibody mechanical systems. However, the existence of joint clearance is inevitable, due to the errors caused by fabrication and assembly of the mechanical components. It not only increases the vibration, noise and wear rate, but also brings undesired effects on the motion accuracy and dynamic behavior of the mechanism [2,3,4,5,6,7,8,9,10]. In addition, high-precision detection of joint clearance is a prerequisite for analyzing and compensating the motion error caused by the joint clearance. Therefore, real-time detection of the micro-clearance in spherical joints is essential to reveal and improve the real dynamic performance of the mechanical systems. 

Many approaches have been proposed for the clearance or displacement measurement, e.g., magnetic sensor [11], inductive sensor [12], capacitive sensor [13], etc. In the case of the magnetic sensor, the displacement is obtained by measuring the variations of the magnetic flux [14]. However, the drawback of the magnetic sensor includes nonlinearity, remanence and temperature dependence [15]. Another method is to deploy the inductive sensors, which have a dual-coil structure, i.e., transmitter and receiver coils. The target displacement is calculated by measuring the voltage variation of the receiver coil induced by the eddy current from the transmitter coil [16,17,18,19]. Compared with the inductive sensor, the capacitive sensor has the advantages of good performance and high accuracy. Ahn [20] proposed a cylindrical capacitive sensor to measure the radial motion of rotating machinery. The radial motion is obtained by detecting the total radial capacitance. Hu et al. [21] proposed a capacitive sensor to capture the clearance of a spherical joint. The capacitive sensor includes a spherical plate and six small arc plates. A point capacitance model is deduced to calculate the clearance from the measured capacitance values and validated by the simulation using Maxwell software. However, there is an average deviation of 10%–13% between the theoretical and simulated capacitance values. Wang et al. [22] proposed a spherical differential capacitive sensor to detect the clearance of a precision spherical joint. The spherical differential capacitive sensor includes eight spherical plates and a ball. The clearance of spherical joints is obtained by measuring the differential capacitance of eight capacitors. However, the capacitive fringe effect, caused by the divergence of the electric field line at the corners of the capacitive plates, could produce additional capacitance and thus lead to the measurement errors [23,24].

To reduce the capacitive fringe effect, this work further proposes an improved capacitive sensor to measure the micro-clearance of a spherical joint. First, the structural design and working principle is presented in Section 2. Then, the mathematical model of the proposed detection method is deduced in Section 3. Finally, the capacitive fringe effect and the sensor performance are simulated using Ansoft Maxwell 16.0 in Section 4 and the theoretical and simulated results are further discussed in Section 5.

## 2. Detecting Method

### 2.1. Structural Design

**Figure 1:**
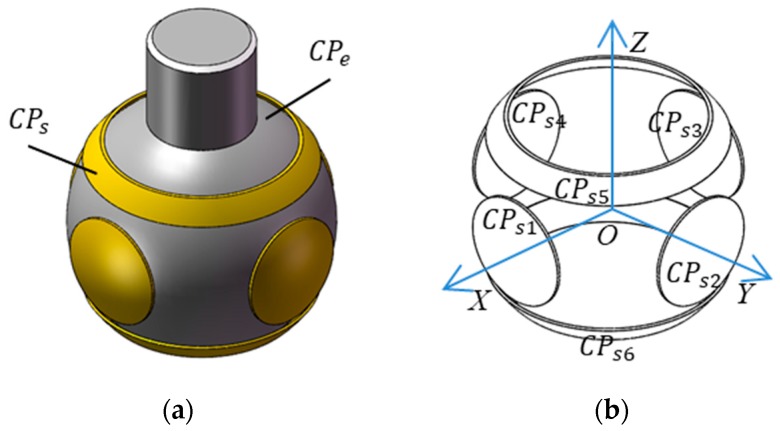
Structural model of the proposed capacitive sensor (**a**) structural assembly (**b**) distribution of the capacitive plates.

**Figure 2:**
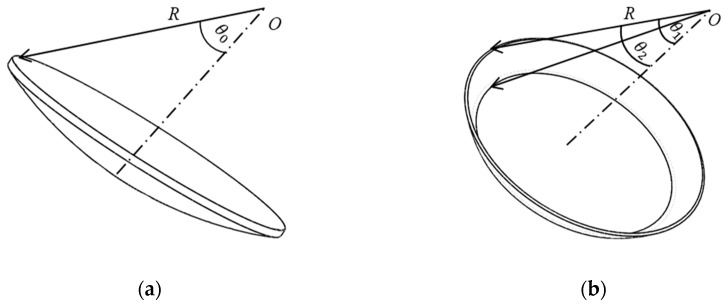
Sketch of the sensing plates (**a**) *CP_s_*_1_ ~ *CP_s_*_4_ (**b**) *CP_s_*_5_ and *CP_s_*_6_.

The proposed capacitive sensor consists of a ball and six spherical plates (Figure 1). The former is the excitation plate (*CP_e_*) while the latter are sensing plates (*CP_si_*, *i* = 1, 2, …, 6). The six sensing plates have the same thickness and area. They are concentrically distributed around the ball. As a result, the sphere center of six spherical plates coincides with the rotational center of the ball. As shown in Figure 1b, the coordinate system *OXYZ* is defined in the sensor with the origin O at the rotational center of the ball, the *X*-axis is chosen along the centerline of plate *CP_s_*_1_ and the *Y*-axis is defined along the centerline of the plate *CP_s_*_2_. In this work, the proposed sensing plates have a structure of the spherical cap (Figure 2). The central angle subtended from the apex of the cap to the edge of the cap for the plates *CP_s_*_1_ *~ CP_s_*_4_ is *θ*_0_, while that for the plates *CP_s_*_5_ and *CP_s_*_6_ is *θ*_1_ and *θ*_2_, due to the motion requirement of the output rod of spherical joints. To achieve the insulation between the sensing plates and the excitation plate, a dielectric material with lubricating and abrasion-resistant properties is deposited on the inner surface of the sensing plates.

### 2.2. Measuring Principle

In the proposed capacitive sensor, the ball of the spherical joint is employed as a common excitation plate. Each sensing plate (*CP_si_*, *i* = 1, 2, …, 6) and the common excitation plate (*CP_e_*) produce a capacitor (*C_i_*, *i* = 1, 2, …, 6). Ideally, the clearance between the ball and six sensing plates is identical, and thus the capacitance value of the capacitor is equal to each other. Once the ball has an eccentric displacement, the center of the ball deviates from the sphere center of six sensing plates. As a result, the clearance between the ball and six sensing plates undergoes different variations, and the capacitance value of each capacitor alters correspondingly. Thus, the eccentric displacement of the ball in the socket could be obtained by detecting the variation of the capacitance values of six capacitors. 

To detect the eccentric displacement of the ball, three capacitor pairs (*C_x_*, *C_y_* and *C_z_*) are established for the measurement of the displacement components along three orthogonal directions (*X*-axis, *Y*-axis and *Z*-axis), respectively. The eccentric displacement (*δ_x_*) of the ball along the *X*-axis can be calculated from the differential capacitance (Δ*C_x_*) of the capacitor pair (*C_x_*) along the *X*-axis, which consists of the capacitor *C*_1_ and the capacitor *C*_3_. Similarly, the eccentric displacement (*δ_y_*) of the ball along the *Y*-axis can be obtained from the differential capacitance (Δ*C_y_*) of the capacitor pair (*C_y_*) along the *Y*-axis, which includes the capacitor *C*_2_ and the capacitor *C*_4_. In addition, the eccentric displacement (*δ_z_*) of the ball along the *Z*-axis can be calculated from the differential capacitance (Δ*C_z_*) of the capacitor pair (*C_z_*) along the *Z*-axis, which comprises of the capacitor *C*_5_ and the capacitor *C*_6_. Thus, the eccentric displacement components (*δ_x_*, *δ_y_* and *δ_z_*) of the ball can be expressed by the following equations:

(1) $$\delta_{x}=f_{x}\Delta C_{x}=f_{x}\left(C_{1}-C_{3}\right)$$

(2) $$\delta_{y}=f_{y}\Delta C_{y}=f_{y}\left(C_{2}-C_{4}\right)$$

(3) $$\delta_{z}=f_{z}\Delta C_{z}=f_{z}\left(C_{5}-C_{6}\right)$$

where *f_x_*, *f_y_* and *f_z_* are the mathematical functions for the eccentric displacement component and the corresponding differential capacitance along the *X*-axis, *Y*-axis and *Z*-axis, respectively. 

## 3. Mathematical Model

To simplify the calculation of the capacitance value of each capacitor, two assumptions are made as follows: (1) the area element *dA* of each spherical-plate capacitor is assumed to be the parallel-plate capacitor with a uniform gap; (2) the fringe effect of the capacitive plate is neglected. Thus, the capacitance value of each capacitor can be given by the following equation:

(4) $$C_{i}=\varepsilon \iint_{A_{i}}\frac{1}{d}dA\;\left(i=1,2,\ldots ,6\right)$$

where is *ε* is the permittivity of the dielectric material, *d* is the clearance between the sensing plate and the ball, *A_i_* is the area of the sensing plate *CP_si_*, *i* = 1, 2, …, 6.

### 3.1. Clearance between the Sensing Platees and the Ball

**Figure 3:**
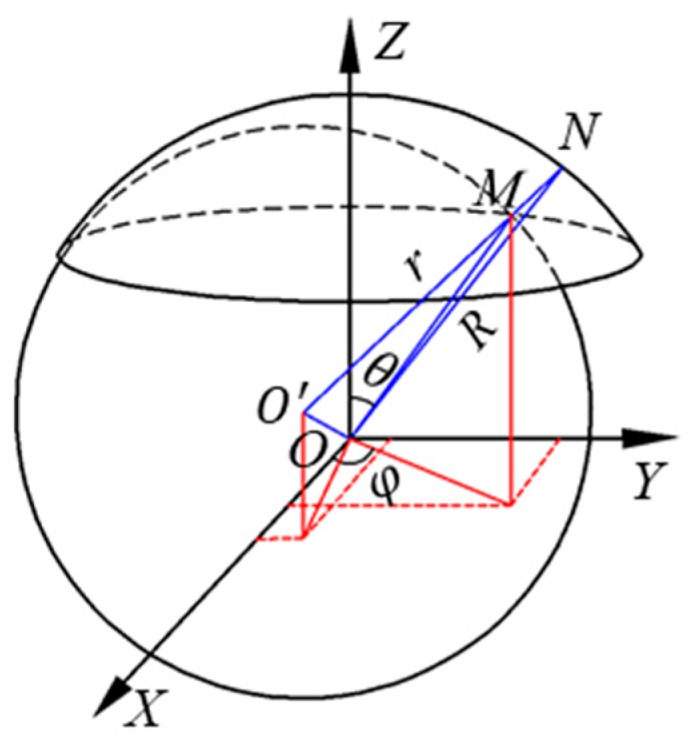
Sketch of the clearance between the ball and the sensing plates.

Figure 3 presents the schematic model for calculating the eccentric displacement of the ball. The surfaces of the sensing plates and the ball are assumed to be ideal spheres. The sphere center of sensing plates coincides with the origin *O* of the coordinate system *OXYZ*, and the center of the ball is denoted by the point *O′*. *δ* is the eccentric displacement from the center *O′* of the ball to the sphere center *O* of sensing plates. The point *M* locates on the surface of the ball. *θ* is the angle between the line *OM* and the *Z*-axis. *φ* is the angle between the line *OM* and the *X*-axis. *N* is the intersection point of the extension of the line *O′M* and the inner surface of sensing plates. *R* is the inner radius of the sensing plates and *r* is the radius of the ball. Thus, the clearance *d* between the ball and the sensing plates at the point *M* is denoted by the distance from point *M* to point *N*. It can be expressed by the following equation:

(5) $$d=R-\delta_{x}\sin\theta \cos\varphi -\delta_{y}\sin\theta \sin\varphi -\delta_{z}\cos\theta -r$$

### 3.2. Dependence of Differential Capacitance on Eccentric Displacement

At the initial position, the ball has no eccentric displacements relative to the spherical sensing plates, the null clearance between the ball and the sensing plate is denoted by *d_0_* = *R − r*. To facilitate the mathematical deduction, four dimensionless parameters are set: *h* = *d/d_0_*, *λ_x_* = *δ_x_/d_0_*, *λ_y_ = δ_y_/d_0_, λ_z_ = δ_z_/d_0_*. Then, Equation (5) can be rewritten into dimensionless form:

(6) $$h=1-\lambda_{x}\sin\theta \cos\varphi -\lambda_{y}\sin\theta \sin\varphi -\lambda_{z}\cos\theta$$

Let $\lambda =\lambda_{x}\sin\theta \cos\varphi +\lambda_{y}\sin\theta \sin\varphi +\lambda_{z}\cos\theta$, we have λ<1 since the eccentric displacement of the ball is smaller than the null clearance. Thus, 1/*h* can be expanded into Maclaurin series as follows:

(7) $$\frac{1}{h}=\frac{1}{1-\lambda }=1+\lambda +\lambda^{2}+\lambda^{3}+\cdots$$

Substituting Equations (6) and (7) into Equation (4), the capacitance value of the capacitor can be expressed by:

(8) $$C_{i}=\frac{\varepsilon }{d_{0}}\iint_{A_{i}}G(\theta ,\varphi )dA\;(i=1,\;2,\;\ldots ,\;6)$$

where $G(\theta ,\varphi )=\sum_{n=0}^{\infty }(\lambda_{x}\sin\theta \cos\varphi +\lambda_{y}\sin\theta \sin\varphi +\lambda_{z}\cos\theta {)}^{n}$, $dA=R^{2}\sin\theta d\theta d\varphi$.

The eccentric displacement of the ball along Z-axis can be obtained by calculating the capacitance difference Δ*C_z_*, which can be obtained from capacitance value of capacitors *CP*_s5_ and *CP*_s6_. Note that the central angles subtended from the apex of the cap to two edges of the cap for *CP*_s5_ and *CP*_s6_ are *θ*_1_ and *θ*_2_. To calculate the integral in Equation (8), the integral interval of the variables *θ* and *φ* can be expressed as follows: 0 ≤ *φ* ≤ 2π and *θ*_1_ ≤ *θ* ≤ *θ*_2_ for *CP*_S5_, 0 ≤ *φ* ≤ 2π and π−*θ*_2_ ≤ *θ* ≤π−*θ*_1_ for *CP*_s6_. Thus, the capacitance value of capacitors *C*_5_ and *C*_6_ can be given by:

(9) $$C_{5}=\frac{\varepsilon }{d_{0}}\int_{\theta_{1}}^{\theta_{2}}\int_{0}^{2\pi }G\left(\theta ,\varphi \right)R^{2}\sin\theta d\theta d\varphi$$

(10) $$C_{6}=\frac{\varepsilon }{d_{0}}\int_{\pi -\theta_{2}}^{\pi -\theta_{1}}\int_{0}^{2\pi }G\left(\theta ,\varphi \right)R^{2}\sin\theta d\theta d\varphi$$

Since the rotational angle of spherical joints in most practical applications is in the range of −15°–15° [25], let *θ*_1_ = 35° and *θ*_2_ = 45°. Then, Equations (9) and (10) can be rewritten by neglecting the terms of higher order beyond the fifth order:

(11) $$C_{5}=\frac{\varepsilon R^{2}}{d_{0}}F_{5}\left(\lambda_{x},\lambda_{y},\lambda_{z}\right)$$

(12) $$C_{6}=\frac{\varepsilon R^{2}}{d_{0}}F_{6}\left(\lambda_{x},\lambda_{y},\lambda_{z}\right)$$

where

(13) $$\begin{array}{cc} F_{5}\left(\lambda_{x},\lambda_{y},\lambda_{z}\right)= & 0.8390+0.5372\lambda_{z}+\ldots \\ & \;0.3453\lambda_{z}{}^{2}+0.2469\left(\lambda_{x}{}^{2}+\lambda_{y}{}^{2}\right)+\ldots \\ & \;0.2227\lambda_{z}{}^{3}+0.4718\lambda_{z}\left(\lambda_{x}{}^{2}+\lambda_{y}{}^{2}\right)+\ldots \\ & \;0.1441\lambda_{z}{}^{4}+0.1097{\left(\lambda_{x}{}^{2}+\lambda_{y}{}^{2}\right)}^{2}+0.6034\lambda_{z}{}^{2}\left(\lambda_{x}{}^{2}+\lambda_{y}{}^{2}\right)+\ldots \\ & \;0.0936\lambda_{z}{}^{5}+0.3478\lambda_{z}{\left(\lambda_{x}{}^{2}+\lambda_{y}{}^{2}\right)}^{2}+0.6454\lambda_{z}{}^{3}\left(\lambda_{x}{}^{2}+\lambda_{y}{}^{2}\right) \end{array}$$

(14) $$\begin{array}{ll} F_{6}\left(\lambda_{x},\lambda_{y},\lambda_{z}\right)= & 0.8390-0.5372\lambda_{z}+\ldots \\ & \;0.3453\lambda_{z}{}^{2}+0.2469\left(\lambda_{x}{}^{2}+\lambda_{y}{}^{2}\right)-\ldots \\ & \;0.2229\lambda_{z}{}^{3}-0.4718\lambda_{z}\left(\lambda_{x}{}^{2}+\lambda_{y}{}^{2}\right)+\ldots \\ & \;0.1441\lambda_{z}{}^{4}+0.1097{\left(\lambda_{x}{}^{2}+\lambda_{y}{}^{2}\right)}^{2}+0.6034\lambda_{z}{}^{2}\left(\lambda_{x}{}^{2}+\lambda_{y}{}^{2}\right)-\ldots \\ & \;0.0936\lambda_{z}{}^{5}-0.3478\lambda_{z}{\left(\lambda_{x}{}^{2}+\lambda_{y}{}^{2}\right)}^{2}-0.6454\lambda_{z}{}^{3}\left(\lambda_{x}{}^{2}+\lambda_{y}{}^{2}\right) \end{array}$$

Using Equations (11)–(14), we obtain the differential capacitance Δ*C_z_* along the *Z*-axis,

(15) $$\Delta C_{z}=C_{5}-C_{6}=\frac{\varepsilon R^{2}}{d_{0}}\Delta F_{z}$$

where

(16) $$\begin{array}{cc} \Delta F_{z}= & 1.0744\lambda_{z}+0.4454\lambda_{z}{}^{3}+0.5426\lambda_{z}\left(\lambda_{x}{}^{2}+\lambda_{y}{}^{2}\right)+\ldots \\ & \;\;0.1872\lambda_{z}{}^{5}+0.6956\lambda_{z}{\left(\lambda_{x}{}^{2}+\lambda_{y}{}^{2}\right)}^{2}+1.2908\lambda_{z}{}^{3}\left(\lambda_{x}{}^{2}+\lambda_{y}{}^{2}\right) \end{array}$$

Similarly, the differential capacitance Δ*C_x_* and Δ*C_y_* along the *X* and *Y* axes can be obtained. To avoid the structural interference between six sensing plates, the central angle *θ* for *CP*_s1_~*CP*_s4_ ranges from 0° to 30°. Then, the differential capacitance Δ*C_x_* and Δ*C_y_* along the *X* and *Y* axes can be given by,

(17) $$\Delta C_{x}=C_{1}-C_{3}=\frac{\varepsilon R^{2}}{d_{0}}\Delta F_{x}$$

(18) $$\Delta C_{y}=C_{2}-C_{4}=\frac{\varepsilon R^{2}}{d_{0}}\Delta F_{y}$$

where

(19) $$\begin{array}{cc} \Delta F_{x}= & 1.5708\lambda_{x}+1.3745\lambda_{x}{}^{3}+0.2945\lambda_{x}\left(\lambda_{y}{}^{2}+\lambda_{z}{}^{2}\right)+\ldots \\ & \;1.2108\lambda_{x}{}^{5}+0.06136\lambda_{x}{\left(\lambda_{y}{}^{2}+\lambda_{z}{}^{2}\right)}^{2}+0.8181\lambda_{x}{}^{3}\left(\lambda_{y}{}^{2}+\lambda_{z}{}^{2}\right) \end{array}$$

(20) $$\begin{array}{cc} \Delta F_{y}= & 1.5708\lambda_{y}+1.3745\lambda_{y}{}^{3}+0.2945\lambda_{y}\left(\lambda_{x}{}^{2}+\lambda_{z}{}^{2}\right)+\ldots \\ & \;1.2108\lambda_{y}{}^{5}+0.06136\lambda_{y}{\left(\lambda_{x}{}^{2}+\lambda_{z}{}^{2}\right)}^{2}+0.8181\lambda_{y}{}^{3}\left(\lambda_{x}{}^{2}+\lambda_{z}{}^{2}\right) \end{array}$$

It can be observed from Equations (15)–(20) that the differential structure could eliminate the even-order terms of the eccentric displacement of the ball. This could improve the sensitivity of the capacitive sensor and reduce the nonlinear error. 

According to Equations (15)–(20), the high-order terms of the differential capacitance have the coupling of the displacements along three orthogonal directions (i.e., *X*-axis, *Y*-axis and *Z*-axis) if the ball has an eccentric displacement along three orthogonal directions. This may lead to the nonlinear error of the calculated capacitance. To illustrate the effect of high-order terms, the maximum nonlinear errors of differential capacitances are examined. *E_x_*, *E_y_* and *E_z_* denote the maximum nonlinear errors caused by the high-order terms for Δ*C_x_*, Δ*C_y_* and Δ*C_z_*, respectively. They can be rewritten as follows:

(21) $$\left\{ \begin{array}{c} E_{x}=E_{y}=max(\frac{\Delta F_{x}-1.5708\lambda_{x}}{1.5708\lambda_{x}})=max(\frac{\Delta F_{y}-1.5708\lambda_{y}}{1.5708\lambda_{y}})\\ E_{z}=max(\frac{\Delta F_{z}-1.0744\lambda_{z}}{1.0744\lambda_{x}}) \end{array}\right.$$

**Figure 4:**
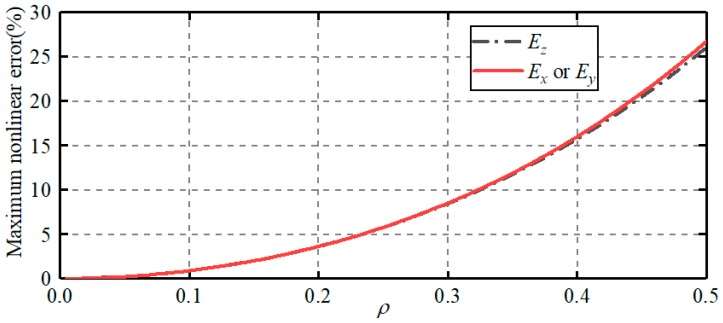
Dependence of the maximum nonlinear errors on the eccentricity of the ball.

Figure 4 presents the dependence of the maximum nonlinear error on the eccentricity of the ball. The eccentricity of the ball *ρ* is defined by $\rho =\sqrt{\lambda_{x}{}^{2}+\lambda_{y}{}^{2}+\lambda_{z}{}^{2}}$. As shown in Figure 4, the maximum nonlinear errors caused by the high-order terms exhibit a quasi-exponential increase with the rising eccentricity of the ball. The maximum nonlinear errors are of 0.88% and 3.62% at the eccentricity *ρ* = 0.1 and *ρ* = 0.2, respectively. Thus, the effect of the high-order terms on the differential capacitance can be neglected, provided that the eccentricity of the ball is small. As such, the dependence of the differential capacitances (Δ*C_x_*, Δ*C_y_* and Δ*C_z_*) on the eccentric displacements (*δ_x_*, *δ_y_* and *δ_z_*) can be described by linear functions,

(22) $$\left\{ \begin{array}{l} \delta_{x}=\frac{d_{0}{}^{2}}{1.5708\varepsilon R^{2}}\Delta C_{x}\\ \delta_{y}=\frac{d_{0}{}^{2}}{1.5708\varepsilon R^{2}}\Delta C_{y}\\ \delta_{z}=\frac{d_{0}{}^{2}}{1.0744\varepsilon R^{2}}\Delta C_{z} \end{array}\right.$$

### 3.3. The Capacitive Fringe Effect Analysis

The capacitive plates of sensors have a limited size in practical applications, the electric field lines diverge at the edges of the capacitive plates and the charge density is higher at the edges or tips, resulting in non-uniform distribution of electric charge. This phenomenon is referred as the capacitive fringe effect. It may increase the capacitance value of a capacitor and thus have a great impact on the detecting accuracy of capacitive sensors. Thus, the capacitance value of a capacitor can be expressed as follows,

(23) $$C=C_{T}+C_{E}$$

where *C* is the actual capacitance value, *C_T_* is the theoretical capacitance value obtained from Equation (4), *C_E_* is the additional capacitance value caused by capacitive fringe effect. Then, the capacitance error *η* caused by the fringe effect is defined as follows,

(24) $$\eta =\frac{C-C_{T}}{C_{T}}$$

## 4. Simulation Setup

In this work, the capacitance values of the capacitive sensor are simulated using ANSOFT Maxwell. Two simulations were carried out. The effect of the plate structure on the capacitive fringe effect was examined first. Then, the relation between eccentric displacement of the ball and differential capacitance of the sensor was investigated in detail.

**Figure 5:**
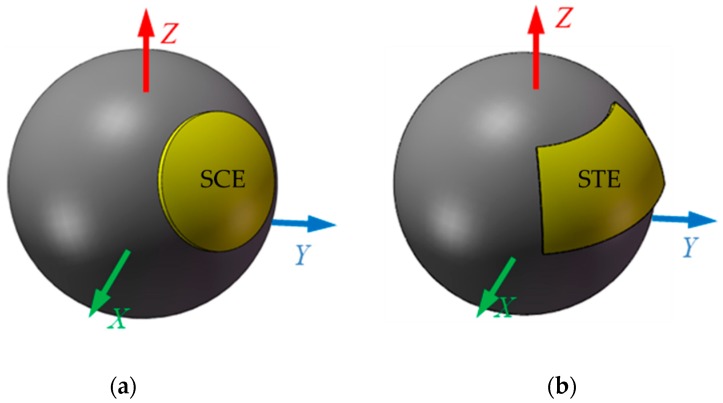
Simulation model of spherical capacitive plate (**a**) SCE: spherical-cap plate (**b**) STE: spherically-trapezoid plate.

To explore the effect of the plate structure on the capacitive fringe effect, two spherical capacitive plates are examined. The spherical-cap capacitive plate proposed in this work is shown in Figure 5a while the spherically-trapezoid capacitive plate proposed in [22] is presented in Figure 5b. The central angle of the spherical-cap capacitive plate is of 30°. The spherical radius of the inner surface for both capacitive plates is of 25 mm and the effective area of two plates is equal to each other. The material of the capacitive plates is copper and the dielectric material is air. The size of simulation domain is four times that of the model to fully reflect the effect of the fringe effect on the capacitance.

**Figure 6:**
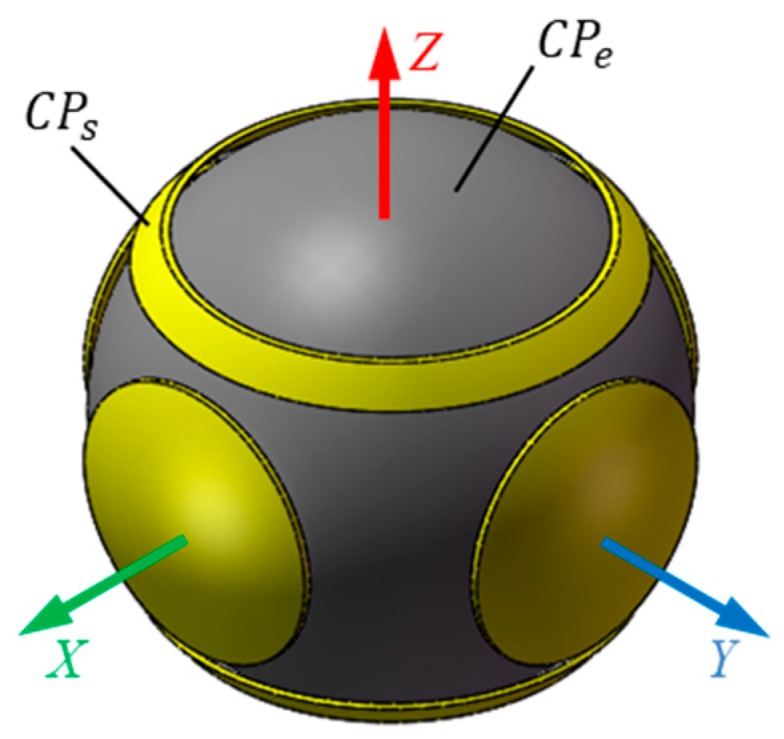
Simulation model of the proposed capacitive sensor.

To explore the relation between the differential capacitance and the eccentric displacement, a simulation model of the proposed capacitive sensor is built using ANSOFT Maxwell (Figure 6). The spherical radius and thickness of the capacitive plates is of 25 mm and 0.5 mm, respectively. The radius of the ball is of 24.8 mm. The null clearance between the ball and the sensing plates is of 0.2 mm. The central angle subtended by the spherical cap for *CE_s_*_1_~*CE_s_*_4_ ranges from 0° to 30° while that for *CE_s_*_5_ and *CE_s_*_6_ is in the range of 35°–45°. Copper is adopted as the material of the excitation and sensing plates, and air is applied as the dielectric material. In the simulation, the eccentric displacement of the ball varies from −40 to 40 µm, with a step of 5 µm. 

## 5. Experimental Setup

**Figure 7:**
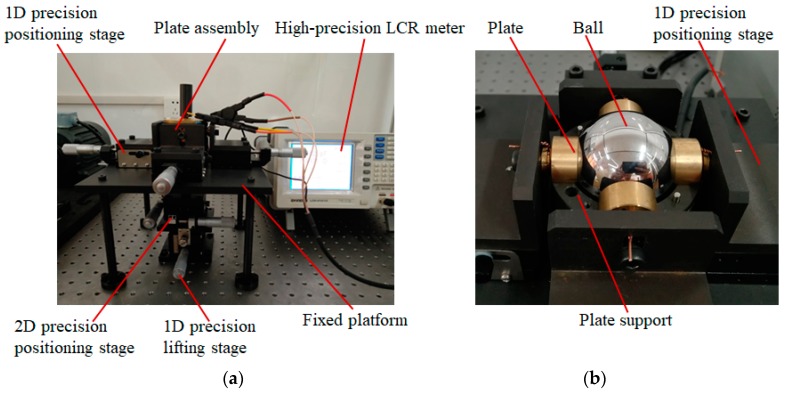
Experimental setup (**a**) Photograph of the test rig, (**b**) photograph of plate assembly.

In this work, an experimental investigation is carried out to validate the feasibility of the proposed method. The experimental setup mainly consists of one-dimensional (1D) precision positioning stage, two-dimensional (2D) positioning stage, 1D precision lifting stage, plate assembly, fixed platform and high-precision LCR meter (Figure 7a). The plate assembly includes six plates, a ball and two plate supports (Figure 7b). The 1D precision positioning stages are attached to plates, while the 2D positioning stage and 1D precision lifting stage are connected to the ball. The material of the plate and the plate supports is copper and aluminum, respectively. The surfaces of the plate supports are hard oxidized to ensure the insulation between the plates and the plate supports.

The concentric arrangement of six capacitive plates is a prerequisite for the detection of the eccentric displacement. In order to make six plates mounted concentrically, a standard steel ball is used as a reference. It has a radius of 25 mm, which equals to the inner radius of the plates. Initially, the standard ball is placed on the plate *CP_S_*_6_. Then, the plates *CP_S_*_1_ ~ *CP_S_*_4_ are attached to the standard steel ball by adjusting four one-dimensional (1D) precision positioning stages. Finally, the plate *CP_S_*_5_ is adjusted to fit the steel ball. A multimeter with its working mode set to the ohmic file is used to detect the contact between the plate and the standard ball. Once six plates are attached to the standard ball, they are concentric about the center of the ball and the position of each plate is recorded.

Another steel ball with a radius of 24.8 mm is used to achieve eccentric displacement. The relative position between the ball and capacitive plates is adjusted by a 2D precision positioning stage and a 1D precision lifting stage. Initially, the ball is positioned at the concentric center of six plates. Then, the ball moves along the direction of *X* = *Y* = *Z* direction, with a step of 10 µm. A high-precision LCR meter (GWINSTEK LCR-8101G) is used to detect the capacitance between the sensing plates and the ball.

## 6. Results and Discussion

### 6.1. Effect of Plate Structure on the Capacitive Fringe Effect

The plate structure has a vital impact on the capacitive fringe effect. In this section, two spherical plates are examined, one is the spherical-cap plate, the other is the spherically-trapezoid plate. The capacitance errors caused by the capacitive fringe effect are investigated in detail at different null clearance and plate thickness. 

**Figure 8:**
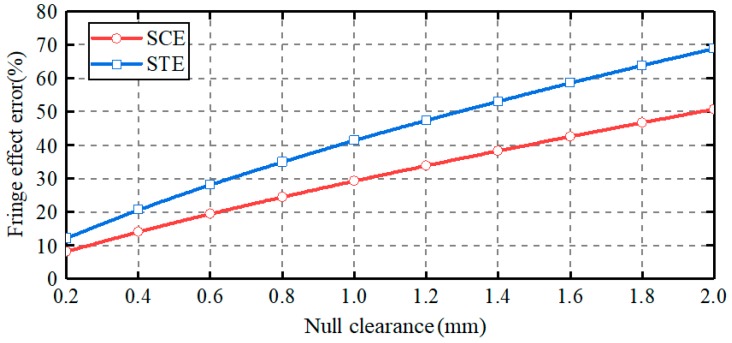
Effect of null clearance on the capacitance errors caused by capacitive fringe effect.

Figure 8 presents the dependence of the capacitance errors on the null clearance between the ball and the sensing plates. The clearance varies from 0.2 to 2 mm, with a step of 0.2 mm. The thickness of the sensing plates is of 2 mm. Two observations can be made. First, for both plates, the capacitance errors caused by the capacitive fringe effect exhibit a remarkable increase with the rising null clearance. In other words, the capacitive fringe effect could be reduced by reducing the null clearance. Second, at the same null clearance, the capacitance error for spherical-cap plate is smaller than that for spherically-trapezoid plate. This indicates that the spherical-cap plate proposed in this work could reduce the capacitive fringe effect, compared with the spherically-trapezoid plate mentioned in [22].

**Figure 9:**
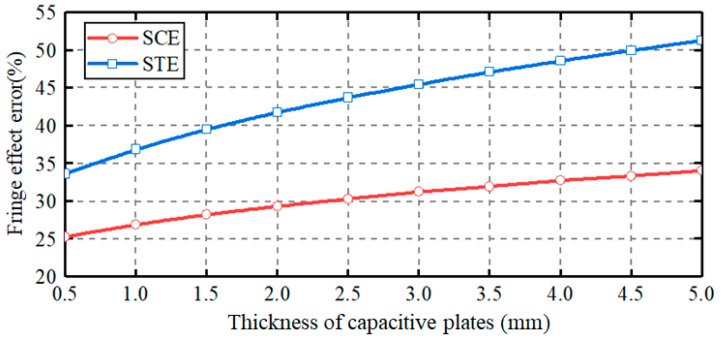
Effect of plate thickness on the capacitance errors caused by capacitive fringe effect.

Figure 9 shows the dependence of the capacitance errors on the plate thickness. The plate thickness varies from 0.5 to 5 mm, with a step of 0.5 mm. The null clearance is fixed at 1 mm. In case of spherical-cap plate, the capacitance error caused by the capacitive fringe effect goes up from 25% to 34% as the plate thickness increases from 0.5 mm to 5 mm. This suggests that the capacitive fringe effect can be reduced by using thin plates. In case of spherically-trapezoid plate, the capacitance error exhibits a larger upward tendency, rising from 33% to 52%, with the increase of the plate thickness. In addition, the capacitance error for spherically-trapezoid plate is larger than that for spherical-cap plate. These indicate that the capacitive fringe effect for spherically-trapezoid plate is more serious and more sensitive to plate thickness. In other words, the spherical-cap plate proposed in this work is beneficial to the reduction of the capacitive fringe effect.

**Figure 10:**
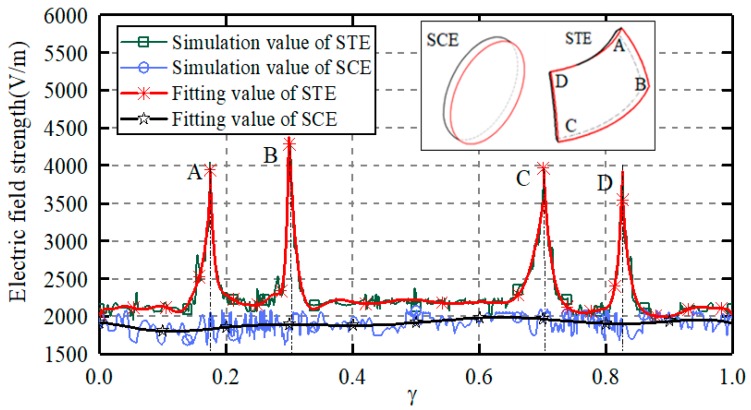
Magnitude distribution of electric field strength along the edge of the capacitive plates.

Figure 10 presents the magnitude distribution of the electric field strength along the edge of the capacitive plates. The theoretical and simulated values are further fitted with a ninth-order polynomial for a better comparison. *γ* is the dimensionless distance normalized by the perimeter of the sensing plate. The clearance between the ball and the sensing plate is of 1.2 mm and the plate thickness is of 2 mm. In case of the spherically-trapezoid plate, the electrons concentrate at the tip of the capacitive plate, resulting an enhanced divergence of the electric field line at the tip. Thus, the electric field strength at the tips A, B, C and D is much larger than that in other places and the capacitive fringe effect becomes more serious. In case of the spherical-cap plate, the electric field strength is relatively uniform along the edge of the capacitive plate. Thus, the proposed capacitive sensor in this work could reduce the capacitive fringe effect and improve the measurement accuracy. 

### 6.2. Characteristics of the Capacitive Sensor

To explore the characteristics of the proposed capacitive sensor, the differential capacitances at different eccentric displacements are investigated in detail. Typical eccentric displacements are examined, that is, displacement along the coordinate axis (*δ_y_* = *δ_z_* = 0, *δ_x_* = *δ_y_* = 0), displacement in the coordinate plane (*δ_x_* = *δ_z_*, *δ_x_* = *δ_y_*) and displacement in the three-dimensional space (*δ_x_* = *δ_y_* = *δ_z_*).

**Figure 11:**
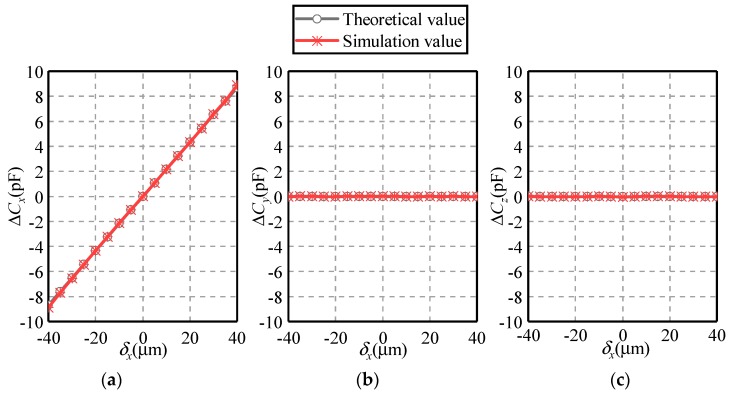
Dependence of the differential capacitance on the eccentric displacement along the *X*-axis (**a**) Δ*C_x_*, (**b**) Δ*C_y_* and (**c**) Δ*C_z_*.

**Figure 12:**
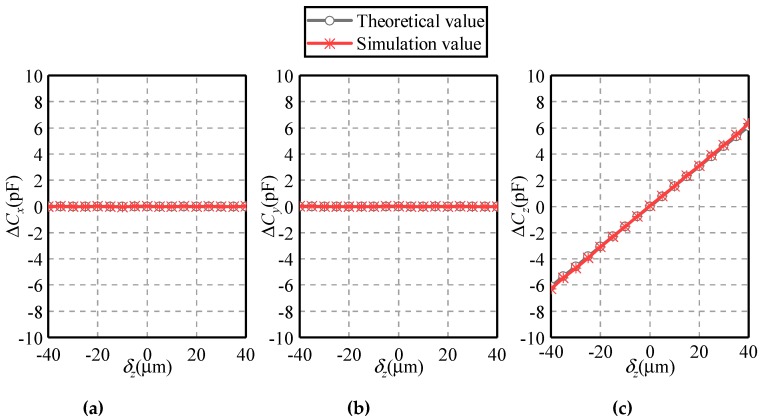
Dependence of the differential capacitance on the eccentric displacement along the *Z*-axis (**a**) Δ*C_x_*, (**b**) Δ*C_y_* and (**c**) Δ*C_z_*.

Figure 11 and Figure 12 present the dependence of the differential capacitances Δ*C_x_*, Δ*C_y_* and Δ*C_z_* on the eccentric displacements along the *X* and *Z* axes, respectively. The eccentric displacement *δ_x_* or *δ_z_* varies from −40 to 40 µm, with a step of 5 µm. The maximal eccentricity of the ball is 0.2. Three observations can be made. First, the simulated values of differential capacitances agree well with their theoretical counterparts, exhibiting a similar variation tendency. This indicates the feasibility and effectivity of the proposed method. Second, if the eccentric displacement is along the *X*-axis, the variation of Δ*C_y_* and Δ*C_z_* exhibits a different trend with that of Δ*C_x_*. As *δ_x_* rises from −40 to 40 µm, Δ*C_x_* goes up linearly from −9 to 9 pF while Δ*C_y_* and Δ*C_z_* remain to zero. This indicates the decoupled effect of the displacement along the three directions, which is consistent with Equations (1)–(3). Third, if the eccentric displacement is along the *Z*-axis, Δ*C_x_* and Δ*C_y_* remain unchanged, while Δ*C_z_* exhibits a linear relationship with *δ_z_*. 

**Figure 13:**
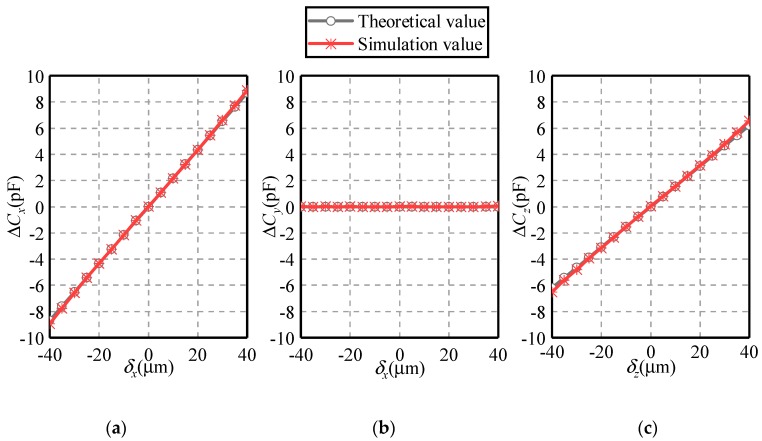
Dependence of the differential capacitance on the eccentric displacement along the *X* = *Z* direction (**a**) Δ*C_x_*, (**b**) Δ*C_y_* and (**c**) Δ*C_z_*.

**Figure 14:**
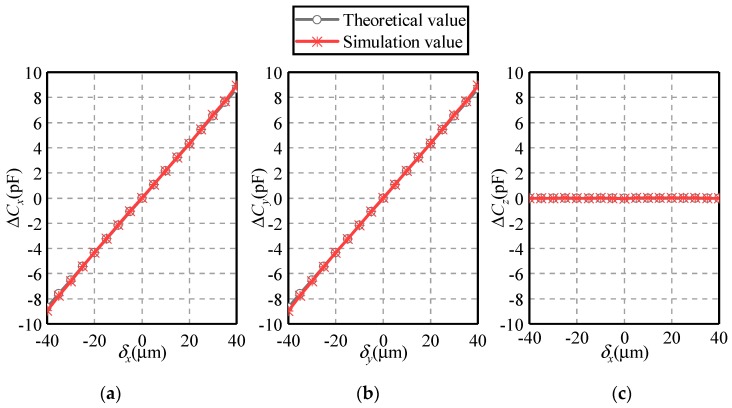
Dependence of the differential capacitance on the eccentric displacement along the *X* = *Y* direction (**a**) Δ*C_x_*, (**b**) Δ*C_y_* and (**c**) Δ*C_z_*.

Figure 13 and Figure 14 show the dependence of the differential capacitances Δ*C_x_*, Δ*C_y_* and Δ*C_z_* on the eccentric displacement along the *X* = *Z* and the *X* = *Y* directions, respectively. The eccentric displacements *δ_x_* = *δ_z_* or *δ_x_* = *δ_y_* vary from −40 to 40 µm, with a step of 5µm. The eccentricity of the ball is from 0 to 0.283. It can be seen that the simulated values of the differential capacitance exhibit a good agreement with the theoretical ones, except a small deviation over ±(30–40) µm. In the case of the eccentric displacement along the *X* = *Z* direction, Δ*C_x_* and Δ*C_z_* rise linearly with the increase of the eccentric displacement, while Δ*C_y_* remains unchanged over the range of −40 µm ≤ *δ_x_* = *δ_z_* ≤ 40 µm. In the case of the eccentric displacement along the *X* = *Y* direction, Δ*C_x_* and Δ*C_y_* exhibit a linear rising trend with the eccentric displacement. 

**Figure 15:**
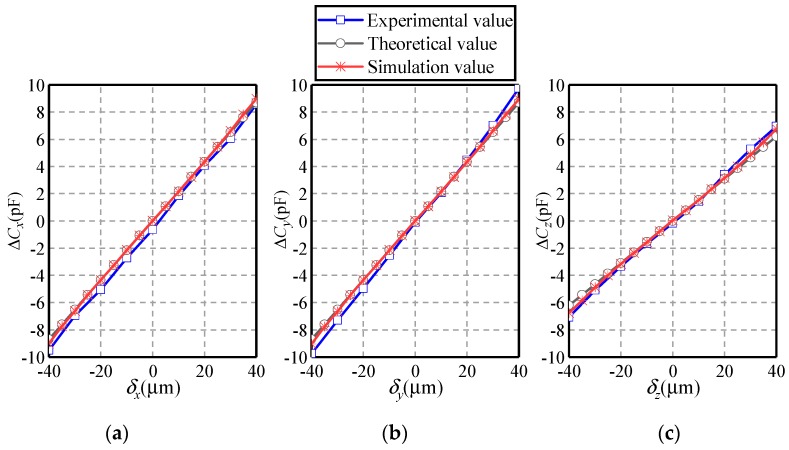
Dependence of the differential capacitance on the eccentric displacement along the *X* = *Y* = *Z* direction. (**a**) Δ*C_x_*, (**b**) Δ*C_y_*, and (**c**) Δ*C_z_*.

Figure 15 presents the dependence of the differential capacitances Δ*C_x_*, Δ*C_y_* and Δ*C_z_* on the eccentric displacement along the *X* = *Y* = *Z* direction. The eccentric displacements *δ_x_* = *δ_y_* = *δ_z_* vary from −40 to 40 µm. The eccentricity of the ball is less than 0.346. Several observations can be made. Firstly, the variation of the simulated values of the differential capacitances exhibit a similar trend with that of the theoretical ones. Secondly, as the eccentric displacement rises from −40 to 40 µm, Δ*C_x_*, Δ*C_y_* and Δ*C_z_* increase linearly. Note that the increasing magnitude of Δ*C_z_* is less than that of Δ*C_x_* and Δ*C_y_*, suggesting that the sensitivity of capacitor pair *C_z_* is less than that of *C_x_* and *C_y_*. Thirdly, the experimental values of the differential capacitances Δ*C_x_*, Δ*C_y_* and Δ*C_z_* present a similar variation tendency with the simulated and theoretical counterparts. This indicates the feasibility of the proposed detection method. Note that the experimental values in Δ*C_x_* exhibit an averaged relative derivation of 10.91% from simulated values and of 10.90% from theoretical values. This can be ascribed to the manufacturing and installation errors of the plates and the ball.

In summary, the theoretical and simulated analysis of the differential capacitances is presented to explore the characteristics of the proposed capacitive sensor. The simulated values of the differential capacitances agree well with their theoretical counterparts, exhibits a linear relation with the eccentric displacement of the ball. This suggests the feasibility of the proposed detection method. More importantly, the spherical-cap plates proposed in this work could greatly reduce the nonlinear error caused by the capacitive fringe effect. This is also reflected by the good agreement between the theoretical and simulated values of the differential capacitances at different eccentric displacements. Besides, the simulated results show that a thin capacitive plate and a small null clearance could contribute to the reduction of the capacitive fringe effect. Note that a common method to avoid the fringing effect is to deploy guard rings. However, this requires the insulation between the guard ring and the capacitive plates and the minimized distance between them. In real applications, these requirements cannot be achieved easily and the effect of the guard ring could be discounted. Compared with our previously proposed sensor with guard ring [22], the present sensor could reduce the fringing effect and achieve a better linearity. This could improve the measurement accuracy of the sensor. 

The sensor proposed in this work consists of a ball and six sensing plates. To achieve the insulation between the capacitive plates, a real joint should have a solid dielectric to separate the plates and it must also be the bearing surface for the ball. This can be done by depositing the dielectric on the surfaces of the sensing plates and the ball. Note that when the dielectric on their surfaces becomes worn, an air gap between the sensing plates and the ball could be widened. In addition, if the dielectric material is totally worn, the sensing plate would contact with the ball and short-circuit will be actuated. In other words, the wear of the dielectric material can be detected. It should be noted that the air gap between the ball and the sensing plates mainly results from the fabrication errors, assembly errors and the requirement of maintaining the relative motion. The eccentric displacement of the ball in the socket will inevitably occur during the operation of spherical joints. For instance, variation in force and torque caused by the motion of the robotic arm and shock transmission could lead to the eccentric displacement of the ball. As a result, there is a variation in the air gap between the ball and each sensing plate. Thus, the air is used as dielectric material in the simulation model.

This work presents a preliminary experimental validation. The similar variation tendency between the experimental and theoretical differential capacitances suggests the feasibility of the proposed sensor. However, there are small deviations between the experimental and theoretical results, which may result from the machining and assembly errors of the plates and the ball. Further studies are still required to improve the accuracy and linearity of the real capacitive sensor. Accurate fabrication and careful examination of the capacitive plates are required before conducting the experiments. Moreover, the machining errors in the ball (e.g., deviation from a sphere) and in the plates (e.g., shape, thickness) should be calibrated. Thus, extensive studies are still required to explore the calibration methods for the machining and assembly errors and to improve the accuracy of a real capacitive sensor.

## 7. Conclusions

This work proposes an improved capacitive sensor for detecting the eccentric displacement of spherical joints. The structure of the capacitive sensor is proposed and the mathematical model of the proposed detection method is deduced. The simulation with Ansoft Maxwell and an experimental investigation are carried out to verify the feasibility of the proposed method. Main conclusions can be made, as follows.

1. The proposed capacitive sensor consists of a ball and six spherical-cap plates. The ball is used as a common excitation plate while six spherical-cap plates are deployed as sensing plates. Each sensing plate and the excitation plate produce a capacitor and every two capacitors with a symmetric distribution form a capacitor pair. The eccentric displacement of the ball can be obtained by detecting the differential capacitances of three capacitor pairs.
2. The mathematical model of the eccentric displacements and the differential capacitances is derived. The nonlinear errors caused by the high-order terms is of 0.88% and 3.62% at the eccentricity of the ball *ρ* = 0.1 and *ρ* = 0.2, respectively. Thus, the relation between the differential capacitances (Δ*C_x_*, Δ*C_y_* and Δ*C_z_*) and the eccentric displacements (*δ_x_*, *δ_y_* and *δ_z_*) can be described by linear function, provided that the eccentricity of the ball is less than 0.2.
3. The capacitance errors caused by the capacitive fringe effect are examined. The capacitive fringe effect could be reduced by reducing the null clearance and plate thickness. In addition, the capacitance error for spherical-cap plate is smaller than that for spherically-trapezoid plate. This indicates that the spherical-cap plate proposed in this work could contribute to the reduction of the capacitive fringe effect, in comparison with the spherically-trapezoid plate.
4. The simulated and experimental values of the differential capacitance agree well with the theoretical counterparts, exhibiting a linear relation between the eccentric displacement and the differential capacitance. This indicates the feasibility and effectivity of the proposed capacitive sensor.
